# Performance of Thromboelastography 6s Difference in Reaction Time Between Heparinase-Free and Heparinase-Containing Channel for Detecting Subtherapeutic Unfractionated Heparin Anticoagulation in a Pediatric Cardiac ICU

**DOI:** 10.1097/CCE.0000000000001400

**Published:** 2026-04-22

**Authors:** Keisuke Nishida, Yusaku Ito, Kota Nakajima, Yusuke Seino, Mana Mitsuguro, Nobuhisa Gommori, Miyako Kyogoku, Madoka Iwahashi, Muneyuki Takeuchi

**Affiliations:** 1 Department of Critical Care Medicine, National Cerebral and Cardiovascular Center, Suita, Osaka, Japan.; 2 Department of Clinical Laboratory, National Cerebral and Cardiovascular Center, Suita, Osaka, Japan.

**Keywords:** anti-Xa activity, diagnostic test performance, heparin, pediatric intensive care unit, thromboelastography

## Abstract

**OBJECTIVES:**

In cases where anti-Xa activity is not routinely measurable, estimating heparin efficacy using the activated partial thromboplastin time (APTT) is challenging, and misestimation may risk bleeding or thrombotic complications. We aimed to assess the utility of the difference in reaction time between thromboelastography 6s heparinase-free and heparinase-containing channel, termed “ΔR,” as a potential diagnostic test for monitoring the heparin effect in a pediatric cardiac ICU (PCICU), using anti-Xa activity as a reference standard, and APTT (seconds and preoperative ratio; APTT at sampling divided by the preoperative value) as comparators.

**DESIGN:**

Single-center, prospective observational diagnostic performance study.

**SETTING:**

The PCICU of a national cardiovascular center in Japan.

**PATIENTS:**

Consecutive patients younger than 18 years old who received unfractionated heparin in the PCICU between July 5, 2024, and May 31, 2025.

**INTERVENTIONS:**

None.

**MEASUREMENTS AND MAIN RESULTS:**

The ΔR, anti-Xa activity, and APTT (seconds and preoperative ratio) were measured for each patient on concurrent samples. Among 59 patients, the ΔR correlated moderately with anti-Xa (*r* = 0.67; *p* < 0.001), whereas correlations between anti-Xa activity and APTT were weak (seconds: *r* = 0.06; *p* = 0.65 and ratio: *r* = 0.24; *p* = 0.066). For the primary target condition (anti-Xa < 0.1 international units/mL), ΔR achieved an area under the curve of 0.934 (95% CI, 0.841–0.996) with an optimal cutoff of 5.6 min (95% CI, 4.35–6.35), yielding 96% sensitivity and 83% specificity, thus outperforming APTT (seconds and ratio; *p* < 0.001).

**CONCLUSIONS:**

ΔR showed a moderate correlation with anti-Xa and high discriminative performance in identifying subtherapeutic anticoagulation, thus supporting its use as a surrogate marker for heparin monitoring in PCICUs. However, further external validation and outcome-based studies are needed to confirm these findings.

**KEY POINTS**

**Question**: Can the difference in reaction time between thromboelastography 6s heparinase-free and heparinase-containing channel (ΔR) serve as a potential surrogate for heparin monitoring?

**Findings**: ΔR correlated with anti-Xa activity and showed high discriminative performance in identifying patients with anti-Xa less than 0.1 international units/mL (area under the curve, 0.934; 5.6-min cutoff: sensitivity 96%, specificity 83%).

**Meaning**: ΔR demonstrated potential to monitor heparin effect, especially for subtherapeutic anticoagulation in settings where anti-Xa activity measurement is unavailable or impractical.

Heparin is commonly administered for antithrombotic management in pediatric cardiac ICUs (PCICUs), which provide perioperative care for pediatric patients undergoing cardiac surgery. Common indications for heparin include management after valve replacement or shunt procedures, for mechanical circulatory support (MCS), and for prevention of catheter-related venous thromboembolism (CVTE). The accurate monitoring of the heparin effect is essential to minimize the risks of bleeding and thrombosis. Assessment of anti-Xa activity is widely regarded as a reference standard for monitoring the heparin effect (1, 2). Nevertheless, only a limited number of institutions in Japan conduct anti-Xa testing. In settings where anti-Xa assessment is not possible, measurement of the activated partial thromboplastin time (APTT) is widely used as an alternative. However, the APTT may be prolonged not only by the anticoagulant effect of heparin but also by deficiencies in coagulation factors (3–5); conversely, acute-phase elevations of fibrinogen or factor VIII can shorten the APTT, resulting in pseudo-heparin resistance (6–9). Consequently, APTT correlates poorly with anti-Xa activity. These features further compromise the interpretability of APTT, making heparin monitoring particularly challenging, and potentially leading to complications. In addition, interpretation of anti-Xa activity can be challenging when analytical interferences are present (e.g., marked hemolysis, lipemia, or hyperbilirubinemia); therefore, an adjunct bedside measure that reflects heparin effect may be clinically useful (10).

TEG 6s is a thromboelastography point-of-care (POC) viscoelastic test used to guide perioperative hemostatic management, allowing comprehensive assessment with multiple reagents. The difference in reaction time (ΔR) between the heparinase-free and heparinase-containing channels (CK and CKH, respectively) can isolate the heparin-dependent delay in clot initiation and has therefore been proposed as a surrogate measure of heparin efficacy (11). However, the utility and diagnostic performance of ΔR for heparin monitoring remain uncertain, limiting its clinical adoption.

Herein, we evaluated the utility of ΔR as a potential diagnostic test for monitoring heparin effect (using anti-Xa activity as a reference standard) and assessed its clinical usefulness by comparing it with conventional APTT metrics (seconds and preoperative ratio).

## MATERIALS AND METHODS

### Study Design, Setting, and Ethical Approval

This study was conducted as a single-center, prospective, observational study in the PCICU of the National Cerebral and Cardiovascular Center, a specialized national institute for patients with cerebral and cardiovascular diseases in Japan. The results are reported following the Standards for Reporting of Diagnostic Accuracy Studies (STARD) reporting guideline (12), and the STARD reporting checklist (13) was applied when editing (the checklist is included in the **Supplemental Digital Content**, https://links.lww.com/CCX/B618).

This study was approved by the Institutional Review Board of the National Cerebral and Cardiovascular Center (approval number: R24029; approval date: July 5, 2024). All procedures were conducted in accordance with the ethical standards of the institutional research committee and the 1975 Helsinki Declaration and its later amendments. Written informed consent for enrollment was obtained from patients or their parents/legal guardians, with assent obtained from children when appropriate.

### Participants (Eligibility and Enrollment)

This study included consecutive patients younger than 18 years admitted to the PCICU of the National Cerebral and Cardiovascular Center after cardiac surgery (both post-cardiopulmonary bypass and non-bypass procedure) or for medical admission between July 5, 2024, and May 31, 2025 (inclusive), who received unfractionated heparin (UFH) for antithrombotic management. Eligible indications for UFH included MCS, extracorporeal membrane oxygenation (ECMO) or ventricular assist device, prophylactic antithrombotic therapy after cardiac surgery (including modified Blalock-Taussig shunt, right ventricle to pulmonary artery conduit, Fontan, Glenn, transannular patch, valve replacement), and prophylaxis for CVTE.

The exclusion criteria were: receipt of antithrombotic agents other than UFH (e.g., warfarin, direct oral anticoagulants, antiplatelet agents), an inability to obtain informed consent, or determination that participation was inappropriate for safety or feasibility reasons by the principal investigator.

Consecutive patients who met eligibility criteria were enrolled throughout the study period. During the study period, 65 eligible patients were identified, and 59 were enrolled in the analysis.

### Index Test, Reference Standard, and Comparators

#### Index Test

ΔR was set as the index test, and was defined as the difference in reaction time (R) between the kaolin channel (CK) and the kaolin plus heparinase channel (CKH): ΔR = CK-R−CKH-R (min). ΔR isolates the heparin-dependent delay in clot initiation.

#### Reference Standard

Anti-Xa activity was prespecified as a reference standard for UFH effect in this study (1, 2). In many MCS protocols, anti-Xa target levels of approximately 0.3–0.7 international units (IU)/mL are commonly applied (2). However, during the pediatric postoperative period, particularly with high-risk bleeding situations such as postcardiotomy ECMO, robust evidence supporting a single therapeutic anti-Xa range remains limited (14). For this population, lower-intensity anticoagulation protocols have been proposed (15). Furthermore, in our PCICU, many UFH indications, such as Fontan/valve-related prophylaxis, CVTE prophylaxis, and some MCS cases with high risk of bleeding, require identification of subtherapeutic anticoagulation rather than confirmation of high-intensity therapeutic anticoagulation. Given that our intended clinical application was bedside screening for subtherapeutic anticoagulation in routine PCICU practice, we prespecified an anti-Xa activity of less than 0.1 IU/mL as the primary diagnostic threshold, with secondary thresholds of less than 0.2 IU/mL and less than 0.3 IU/mL.

#### Comparators

The APTT, reported both in seconds and as the preoperative ratio, was used as a comparator. APTT ratio was calculated as the APTT at sampling divided by the preoperative APTT. Because APTT in pediatric patients is age-dependent, and many exhibit preoperative prolongation due to developmental hemostasis or underlying conditions, we additionally expressed APTT as a ratio to each patient’s baseline value.

#### Sample Collection and Measurements

At each sampling time point, blood was drawn from the arterial catheter, with precautions taken to avoid heparin contamination. Blood was collected simultaneously for all assays and processed concurrently in separate tubes. The following analyses were conducted: TEG 6s Global Hemostasis assay (Haemonetics, Braintree, MA); anti-Xa activity on the ACL TOP 350 CTS (Instrumentation Laboratory, Bedford, MA) using a chromogenic assay (HemosIL Liquid Anti-Xa, Instrumentation Laboratory, Bedford, MA); and APTT on the STACIA CN10 (PHC Corporation, Tokyo, Japan) with the Dade Actin Activated PTT Reagent (Sysmex Corporation, Kobe, Japan). The sampling time points were scheduled at random by the principal investigator or a co-investigator within routine clinical care. Each patient contributed a single blood sample. The assessors of ΔR were blinded to the results of anti-Xa activity and APTT, while the laboratory personnel measuring anti-Xa and APTT were blinded to ΔR.

### Statistical Analysis

After assessing normality, continuous variables were summarized as the mean ± sd when approximately normal, or as the median (interquartile range) otherwise. Dichotomous variables were reported as proportions, and multicategory variables as counts.

For primary analysis to assess the association between ΔR and anti-Xa activity, we calculated the Pearson correlation coefficients (*r*). For secondary analyses to assess the association between ΔR and anti-Xa activity, we first fit a simple linear regression of anti-Xa on ΔR (anti-Xa = β_0_ + β_1_·ΔR), reporting the regression coefficients, *R*^2^, and residual sd; and second, compared the dependent correlations *r* (ΔR, anti-Xa) with *r* (APTT, anti-Xa) and *r* (APTT ratio, anti-Xa) using Steiger’s Z test.

For primary diagnostic performance analysis, we constructed receiver operating characteristic (ROC) curves for ΔR, APTT (s), and the APTT ratio for the primary target condition (anti-Xa < 0.1 IU/mL). For these, we reported the area under the curves (AUCs) with 95% CIs, identified optimal cutoffs by Youden’s index, and obtained 95% CIs for AUCs and cutoffs via bootstrap resampling (1000 replicates). Differences in AUCs between the ΔR and APTT (seconds and ratio) were tested using DeLong’s method. For secondary diagnostic analyses, using each marker’s primary optimal cutoff, we reported the sensitivity, specificity, positive predictive value (PPV), and negative predictive value (NPV). The same ROC analyses were individually repeated for the secondary target conditions (anti-Xa < 0.2 and < 0.3 IU/mL).

Because antithrombin deficiency may influence heparin monitoring, we performed a prespecified sensitivity analysis restricting the dataset to measurements with antithrombin activity (AT) greater than or equal to 60% and repeated the primary association analysis and diagnostic performance analyses in the restricted cohort. For the main analysis to assess the association between ΔR and anti-Xa activity (linear regression), an a priori power analysis with an effect size f^2^ = 0.15 (medium), α = 0.05, and power = 0.80 indicated a required sample size of 55. Allowing for an attrition of 15%, the planned sample size was 65. A complete case analysis was performed for which a two-sided *p* value of less than 0.05 was considered statistically significant. Data were analyzed using RStudio (Version 2024.9.1.394, Posit Software PBC, Boston, MA) with R (Version 4.4.0, released on April 24, 2024; R Foundation for Statistical Computing, Vienna, Austria).

## RESULTS

Of the 65 patients, 59 were enrolled in the analysis. A flowchart of patient enrollment/exclusion is shown in **Figure S1** (https://links.lww.com/CCX/B618). Six were excluded, including those in whom heparin was discontinued before sampling. There were no failed measurements, indeterminate results, or missing data. ΔR values were negative in three measurements, and these values were retained as measured in the analyses. The baseline characteristics of the enrolled participants are summarized in **Table 1**. All patients received UFH by continuous infusion without bolus administration; the infusion dose distribution is summarized in Table 1.

**TABLE 1.** Characteristics of the Enrolled Patients (*n* = 59)

Variables	Overall (*n* = 59)
Age (d)	475 (53–1588)
Sex, male (%)	27 (45.8)
Weight (kg)	7.70 (4.55–13.10)
Body surface area (m^2^)	0.37 (0.25–0.64)
Heparin dose (U/kg/hr)	5.40 (4.00–8.95)
Antithrombin activity (%)	79.9 ± 18.6
Heparin reason	
Prevention for catheter-related venous thromboembolism	23
Right ventricle outlet tract reconstruction	8
Blalock-Taussig shunt	9
Pulmonary artery plasty	3
Total cavopulmonary connection	6
Mechanical circulatory support	3
Coronary artery thrombus prevention	1
Intracardiac thrombus prevention	1
Aortic valve replacement	1
Unifocalization	1
Bidirectional Glenn anastomosis	2
Deep vein thrombosis	1

Data are presented as the median (interquartile range) or mean ± sd, and number (proportion).

### Association Between ΔR and Anti-Xa Activity

**Figure 1.**
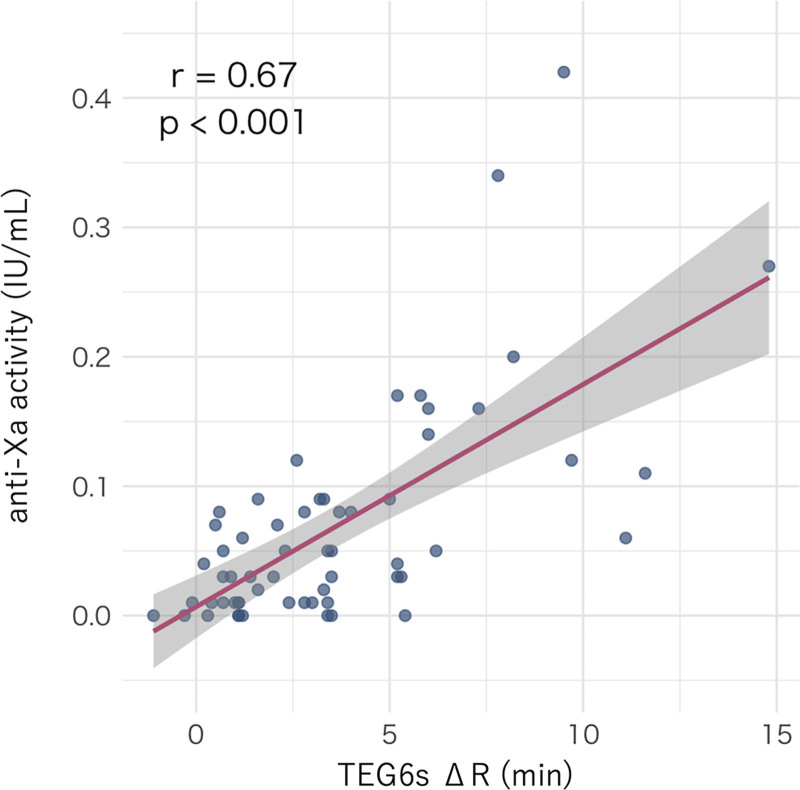
The relationship between difference in reaction time between heparinase-free and heparinase-containing channel (min; ΔR) and anti-Xa activity (*scatter* with *regression line*). Pearson *r* = 0.67 (*p* < 0.001). The simple regression equation was: anti-Xa = 0.0068 + 0.0172 × ΔR (*R*^2^ = 0.4492; residual sd = 0.0623). The *shaded area* indicates the 95% confidence band. IU = international units, TEG, thromboelastography.

**Figure 2.**
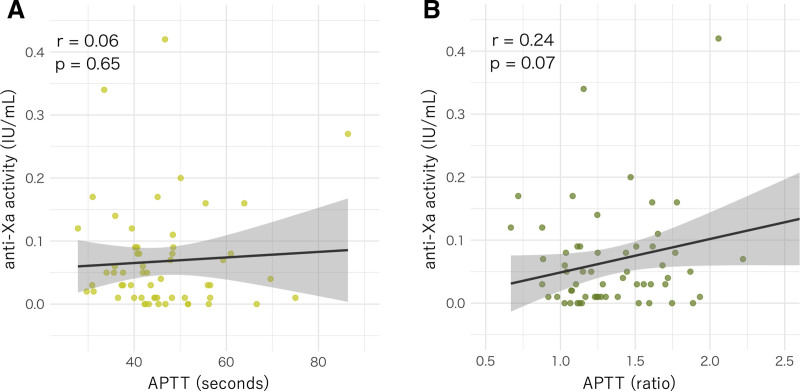
The relationship between activated partial thromboplastin time (APTT) measures and anti-Xa activity (*scatter* with *regression line*). **A**, APTT (s): Pearson *r* = 0.06 (*p* = 0.65); anti-Xa = 0.0476 + 0.0004 × APTT (*R*^2^ = 0.0035; residual sd = 0.0838). **B**, APTT ratio: Pearson *r* = 0.24 (*p* = 0.066); anti-Xa = −0.0043 + 0.0531 × APTT ratio (*R*^2^ = 0.0580; residual sd = 0.0815). *Shaded areas* indicate 95% confidence bands. IU = international units.

ΔR correlated moderately with anti-Xa activity (*r* = 0.67; *p* < 0.001; **Fig. 1**). Simple linear regression yielded the formula anti-Xa = 0.0068 + 0.0172 × ΔR with *R*^2^ = 0.4492 (45% of variance explained) and residual sd = 0.0623. In contrast, APTT showed weak associations with anti-Xa: for APTT (s), *r* = 0.06 (*p* = 0.65) and anti-Xa = 0.0476 + 0.0004 × APTT (*R*^2^ = 0.0035; residual sd = 0.0838); for the APTT ratio, *r* = 0.24 (*p* = 0.066) and anti-Xa = −0.0043 + 0.0531 × APTT ratio (*R*^2^ = 0.0580; residual sd = 0.0815; **Fig. 2**, ***A*** and ***B***). Steiger’s test confirmed that the correlation with anti-Xa was significantly stronger for ΔR than for APTT (seconds: *p* < 0.0001; ratio: *p* = 0.0012).

### Diagnostic Performance Assessment

**Figure 3.**
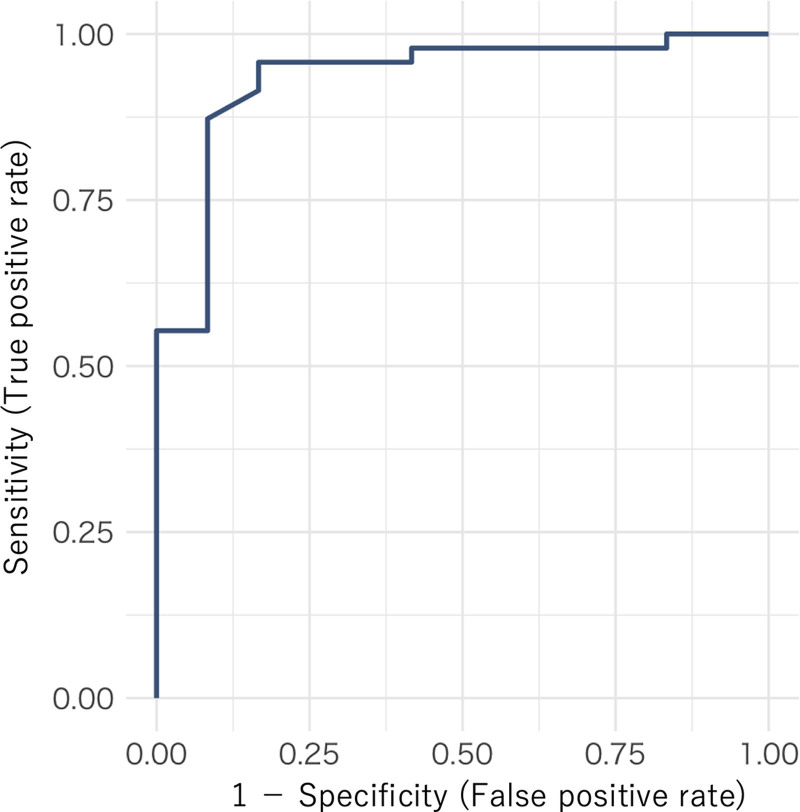
Receiver operating characteristic curve for the ability of difference in reaction time between heparinase-free and heparinase-containing channel (min) to detect anti-Xa activity less than 0.1 international units/mL. Area under the curve, 0.934 (95% CI, 0.841–0.996); Youden’s optimal cutoff 5.6 min (95% CI, 4.35–6.35 min).

ROC analyses for the primary target condition (anti-Xa < 0.1 IU/mL) revealed an AUC of 0.934 for ΔR (95% CI, 0.841–0.996), with an optimal cutoff of 5.6 min (95% CI, 4.35–6.35 min; **Fig. 3**). For the secondary target conditions, the AUCs for ΔR were 0.959 (95% CI, 0.893–1.000) with an optimal cutoff of 7.55 min (95% CI, 6.90–12.95) for less than 0.2 IU/mL and 0.921 (95% CI, 0.842–0.982) with a cutoff of 7.55 min (95%CI, 6.90–8.85 min) for less than 0.3 IU/mL (**Fig. S2**, ***A*** and ***B***, https://links.lww.com/CCX/B618). The corresponding ROC curves, including AUCs and cutoffs for APTT and the APTT ratio, are shown in **Figure S3*A–F*** and **Table S1** (https://links.lww.com/CCX/B618). Based on the DeLong’s test, the AUCs for ΔR significantly exceeded those for APTT (seconds and ratio) for anti-Xa less than 0.1 IU/mL (seconds: 95% CI, 0.225–0.658; ratio: 95% CI, 0.209–0.620; both *p* < 0.001). The differences for anti-Xa less than 0.2 IU/mL and less than 0.3 IU/mL were further detailed in **Table S2** (https://links.lww.com/CCX/B618).

Using the Youden’s cutoffs of ΔR and the APTT (seconds and ratio), sensitivity, specificity, PPV, and NPV for the target conditions are summarized in **Table 2**.

**TABLE 2.** Classification Performance at Youden’s Cutoffs for Difference in Reaction Time Between Heparinase-Free and Heparinase-Containing Channel and Activated Partial Thromboplastin Time (Seconds and Preoperative Ratio) to Detect Anti-Xa Less Than 0.1, Less Than 0.2, and Less Than 0.3 International Units/mL

Test	Cutoff	Target Anti-Xa Activity (International Units/mL)	Sensitivity	Specificity	Positive Predictive Value	Negative Predictive Value
Difference in reaction time between heparinase-free and heparinase-containing channel (min)	5.6	< 0.1	0.96	0.83	0.96	0.83
	7.6	< 0.2	0.95	1.00	1.00	0.57
	7.6	< 0.3	0.91	1.00	1.00	0.29
APTT (seconds)	45	< 0.1	0.57	0.58	0.84	0.26
	46	< 0.2	0.64	0.75	0.97	0.13
	34	< 0.3	0.09	0.50	0.83	0.02
APTT at sampling ÷ preoperative APTT	1.6	< 0.1	0.77	0.42	0.84	0.31
	2.0	< 0.2	0.98	0.50	0.96	0.67
	2.0	< 0.3	0.96	0.50	0.98	0.33

APTT = activated partial thromboplastin time.

Cutoffs were determined by Youden’s index from receiver operating characteristic analyses for each target condition. Sensitivity, specificity, positive predictive value, and negative predictive value are point estimates at those cutoffs. Primary target: < 0.1 international units (IU)/mL; < 0.2 and < 0.3 IU/mL are secondary.

### Sensitivity Analysis

In a prespecified sensitivity analysis restricted to measurements with AT greater than or equal to 60% (*n* = 50), ΔR remained moderately correlated with anti-Xa activity (*r* = 0.66; *p* < 0.001), and simple linear regression yielded anti-Xa = 0.0126 + 0.0168 × ΔR (*R*^2^ = 0.4333; residual sd = 0.0654; **Fig. S4*A***, https://links.lww.com/CCX/B618). For the primary target threshold (anti-Xa < 0.1 IU/mL), ΔR retained high diagnostic performance (AUC, 0.932; 95% CI, 0.827–1.000) with an optimal cutoff of 5.6 minutes (95% CI, 4.35–6.35 min; sensitivity, 0.97; specificity, 0.82; **Fig. S4*B***, https://links.lww.com/CCX/B618).

## DISCUSSION

Overall, in the present study, we found a significant positive linear association between ΔR and anti-Xa activity (*r* = 0.67), with a simple linear regression model with anti-Xa activity as the dependent variable, ΔR explained approximately 45% of the observed variability in anti-Xa activity. These findings support the utility of ΔR as an alternative to anti-Xa activity for measuring the heparin effect. For the primary diagnostic threshold (anti-Xa < 0.1 IU/mL), the ΔR showed high discriminative performance (AUC 0.93), with the optimal cutoff of 5.6 minutes yielding 96% sensitivity and 83% specificity. For the secondary threshold (anti-Xa < 0.2 IU/mL), discriminative performance remained excellent (AUC, 0.96), suggesting potential utility for early detection of subtherapeutic anticoagulation. Although the AUC was also high for anti-Xa less than 0.3 IU/mL, the relatively small number of qualifying cases resulted in a low NPV. As such, larger studies are required to confirm the ability of ΔR to measure the effect of heparin producing an anti-Xa activity of less than 0.3 IU/mL.

Comparatively, the APTT, reported either in seconds or the preoperative ratio, showed no meaningful correlation with anti-Xa. Furthermore, APTT underperformed across the AUCs and classification measures based on ROC analysis. This likely reflects the susceptibility of APTT to nonheparin influences. Mechanistically, UFH potentiates antithrombin-mediated inhibition of both thrombin and factor Xa. Anti-Xa activity and ΔR are designed to more directly reflect the heparin attributable component of anticoagulation, whereas APTT represents a global clotting time influenced by heparin as well as nonheparin determinants of the coagulation pathways, which may underlie the weak APTT–anti-Xa associations observed in our study (16, 17). In pediatric patients with congenital heart disease, chronic cyanosis and developmental immaturity can impair the hepatic synthesis of coagulation factors (18–21), while perioperative hemodilution from fluid administration commonly leads to dilutional coagulopathy. Furthermore, in pediatric MCS, lower circuit flow rates compared to adults can promote stasis and circuit thrombosis, potentially contributing to consumptive coagulopathy (2, 18). Furthermore, in perioperative settings, the acute-phase elevations of fibrinogen and factor VIII can shorten the APTT and produce pseudo-heparin resistance. In support, post-pulmonary endarterectomy patients commonly show APTT–anti-Xa discordance (36% of paired samples), most often with subtherapeutic APTT levels, despite therapeutic/supratherapeutic anti-Xa, with marked postoperative FVIII elevations driving this phenomenon (9). Pseudo-heparin resistance is common in patients undergoing ECMO and CRRT, and the APTT–anti-Xa relation is patient-specific (7). Together, these features decouple APTT from the heparin effect, likely explaining the weak APTT–anti-Xa correlations observed in our cohort, underscoring the value of ΔR as a more heparin-specific index. Indeed, in the AUC comparisons by DeLong’s test, ΔR was significantly superior to APTT (seconds and ratio), particularly for the primary target condition anti-Xa less than 0.1 IU/mL.

ΔR is a POC test parameter that can be rapidly obtained at the bedside, and remains feasible even in settings where anti-Xa measurement is impractical. The rationale for using ΔR is that the CKH channel contains heparinase and is intended to neutralize the effect of heparin, thereby isolating the heparin attributable effects to the clot initiation time. According to the manufacturer’s provided documentation for the TEG 6s heparinase assays, the neutralization performance is specified as the ability to neutralize up to 5.0 IU/mL of UFH. Furthermore, experimental evidence also supports a concentration-dependent relationship between heparin activity and cartridge-based TEG 6s parameters in an ex vivo spiking study using healthy donor blood. TEG 6s CK-R quantified heparin concentration across clinically relevant ranges (0.05–0.5 IU/mL), and classified samples into subtherapeutic/therapeutic/supratherapeutic heparin strata with high discriminative performance (22). Considering this evidence, ΔR has been proposed as a potential indicator of heparin efficacy; its utility and diagnostic performance for heparin monitoring have not been thoroughly evaluated. This study is the first to comprehensively assess both, demonstrating utility and high diagnostic performance. Within our empirical ROC range, ΔR cutoffs of 5.6 min (for anti-Xa < 0.1 IU/mL) and 7.6 min (for < 0.2 IU/mL) performed well and can be used as screening thresholds for subtherapeutic anticoagulation in similar settings. In contrast, for anti-Xa greater than or equal to 0.3 IU/mL, ΔR values inferred by inverting the regression line (anti-Xa = 0.0068 + 0.0172 × ΔR) provided only approximate guidance; therefore, we did not establish diagnostic performance at these levels, and observations above 0.3 IU/mL were sparse. For reference, the approximate ΔR values corresponding to anti-Xa values of 0.1, 0.2, 0.3, and 0.4 IU/mL were 5.4, 11.2, 17.0, and 22.9 minutes, respectively; thus, the targets of 0.5–0.6 IU/mL lay outside our observed range and should be considered reference only, with confirmatory anti-Xa testing guiding decisions whenever available.

In this study, we used a prospective consecutive cohort design with concurrent blood sampling and blinded assessments, enabling the comparison of ΔR, APTT (seconds and ratio) against anti-Xa activity. Both the utility and diagnostic performance were evaluated using STARD-aligned methods (correlation/regression; ROC/AUC with bootstrap CIs; DeLong), with prespecified target conditions (12). However, there are several important limitations to this study. First, this was a single-center study, which may limit the generalizability of the findings. Second, the observations were relatively sparse in the upper anti-Xa range (0.2–0.3 IU/mL), making estimates of discriminative performance at these thresholds unstable; further, residual spectrum bias cannot be excluded. Third, we did not link ΔR-guided management to clinical outcomes in this study. To overcome these limitations, multicenter external validation—particularly enriching the 0.2–0.3 IU/mL range—and pragmatic prospective trials to test whether ΔR-guided protocols improve clinical outcomes are warranted. Fourth, antithrombin deficiency can affect the heparin effect and the measurement of anti-Xa activity and ΔR. In our cohort, only a few patients had AT levels less than 60%. Nevertheless, sensitivity analyses limited to measurements with AT greater than or equal to 60% produced results consistent with the primary analysis, suggesting that moderate AT variability did not significantly affect ΔR performance in this setting. However, generalizability to populations with more frequent clinically significant AT deficiency remains uncertain and warrants further investigation. Fifth, because each patient contributed a single paired measurement at a pragmatically scheduled time point, we could not assess within-patient reproducibility or fully account for nonheparin-related factors that may influence coagulation tests. Sixth, α2-macroglobulin, which inhibits thrombin and is relatively higher in neonates and young infants, may influence the relationship between ΔR and anti-Xa activity. We did not measure α2-macroglobulin in this study; therefore, its potential impact on the relationship should be assessed in future studies.

## CONCLUSIONS

Overall, ΔR showed a moderate, statistically significant linear correlation with anti-Xa activity, suggesting potential as a surrogate for anti-Xa to monitor the heparin effect in the PCICU. Using a ΔR cutoff of 5.6 minutes, subtherapeutic anticoagulation (anti-Xa < 0.1 IU/mL) was identified with higher discriminative performance than with APTT (seconds or ratio). Overall, these results support the use of ΔR as an alternative to anti-Xa activity. However, external validation and studies linking ΔR-guided management to clinical outcomes are needed to support clinical implementation.

## ACKNOWLEDGMENTS

We thank Editage (www.editage.jp) for English language editing.

## Supplementary Material
