# Supplementary material for: Performance of Thromboelastography 6s Difference in Reaction Time Between Heparinase-Free and Heparinase-Containing Channel for Detecting Subtherapeutic Unfractionated Heparin Anticoagulation in a Pediatric Cardiac ICU
Source: Crit Care Explor. 2026 Apr 22;8(4):e1400. doi: 10.1097/CCE.0000000000001400 (PMC13098772; doi:10.1097/CCE.0000000000001400)

Supplemental Digital Content

Performance of TEG 6s ΔR for detecting subtherapeutic unfractionated heparin anticoagulation in a pediatric cardiac intensive care unit: A diagnostic accuracy study

Keisuke Nishida, M.D.[1]; Yusaku Ito, M.D.[1]; Kota Nakajima, M.T.[2]; Yusuke Seino, M.D.[1]; Mana Mitsuguro, M.T., Ph.D.[2]; Nobuhisa Gommori, M.D.[1]; Miyako Kyogoku, M.D., Ph.D.[1]; Madoka Iwahashi, M.D.[1]; Muneyuki Takeuchi, M.D., Ph.D.[1]

[1]Department of Critical Care Medicine, National Cerebral and Cardiovascular Center, Suita, Osaka, Japan

[2]Department of Clinical Laboratory, National Cerebral and Cardiovascular Center, Suita, Osaka, Japan

Corresponding author:

Muneyuki Takeuchi

E-mail: takeuchi muneyuki @ncvc.go.jp

Table of Contents

STARD Reporting Checklist **2**

Table S1 .... **4**

Table S2 .... **5**

Figure S1 .... **6**

Figure S2 .... **7**

Figure S3 .... **8**

Figure S4 .... **9**

The STARD reporting checklist

For checking that diagnostic accuracy study articles can be understood and used by everyone

	Item Description	Location (or reason for not reporting)
**Title or abstract**		
1. Identification as a study of diagnostic accuracy	Identification as a study of diagnostic accuracy using at least one measure of accuracy (such as sensitivity, specificity, predictive values, or AUC).	Title<br>Abstract; Design & Measurements and main results
Abstract		
2. Abstract	Structured summary of study design, methods, results and conclusions (for specific guidance, see STARD for Abstracts).	Abstract
Introduction		
3. Background	Scientific and clinical background, including the intended use and clinical role of the index test.	Introduction; paragraph 1 & 2
4. Objectives	Study objectives and hypotheses.	Introduction; paragraph 3
Methods		
5. Study design	Whether data collection was planned before the index test and reference standard were performed (prospective study) or after (retrospective study).	Materials and Methods; Study Design, Setting, and Ethical Approval
Participants		
6. Eligibility criteria	Eligibility criteria.	Materials and Methods; Participants (eligibility and enrollment)
7. Identifying eligible participants	On what basis potentially eligible participants were identified (such as symptoms, results from previous tests, inclusion in registry).	Materials and Methods; Participants (eligibility and enrollment)
8. Setting, location, and dates	Where and when potentially eligible participants were identified (setting, location and dates).	Materials and Methods; Study Design, Setting, and Ethical Approval
9. Consecutive, random or convenience series	Whether participants formed a consecutive, random or convenience series.	Materials and Methods; Participants (eligibility and enrollment)
Test Methods		
10. Index test & Reference standard	10a. Index test<br>10b. Reference standard	Materials and Methods; Index test & Reference standard
11. Reference standard rationale	Rationale for choosing the reference standard (if alternatives exist).	Introduction; paragraph 1<br>Materials and Methods; Reference standard
12. Index test and reference standard cut-offs or categories	12a. Definition of and rationale for test positivity cut-offs or result categories of the index test, distinguishing prespecified from exploratory.<br>12b. Definition of and rationale for test positivity cut-offs or result categories of the reference standard, distinguishing prespecified from exploratory.	Materials and Methods; Index test & Reference standard & Statistical Analysis
13. Information available to performers or readers of the index test and	13a. Whether clinical information and reference standard results were available to the performers or readers of the index test.	Materials and Methods; Sample collection and measurements

reference standard assessors	13b. Whether clinical information and index test results were available to the assessors of the reference standard.	
Analysis		
14. Analysis methods	Methods for estimating or comparing measures of diagnostic accuracy.	Materials and Methods; Statistical Analysis
15. Indeterminate results	How indeterminate index test or reference standard results were handled.	Materials and Methods; Statistical Analysis
16. Missing data	How missing data on the index test and reference standard were handled.	Materials and Methods; Statistical Analysis
17. Variability	Any analyses of variability in diagnostic accuracy, distinguishing prespecified from exploratory.	Materials and Methods; Statistical Analysis
18. Intended sample size	Intended sample size and how it was determined.	Materials and Methods; Statistical Analysis
Results		
Participants		
19. Participant flow diagram	Flow of participants, using a diagram.	Results; paragraph 1 & Figure S1
20. Baseline characteristics	Baseline demographic and clinical characteristics of participants.	Results; paragraph 1 & Table 1
21a. Participants with and without the target condition	21a. Distribution of severity of disease in those with the target condition.<br>21b. Distribution of alternative diagnoses in those without the target condition	Not applicable
22. Time interval	Time interval and any clinical interventions between index test and reference standard.	Materials and Methods; Sample collection and measurements
Test Results		
23. Index test and reference standard results	Cross tabulation of the index test results (or their distribution) by the results of the reference standard.	Results; Diagnostic accuracy assessment & Figure 3& Figure S2 & Table S2 & Table 2
24. Estimates of accuracy	Estimates of diagnostic accuracy and their precision (such as 95% CIs).	Results; Diagnostic accuracy assessment & Figure 3& Figure S2 & Table S2 & Table 2
25. Adverse events	Any adverse events from performing the index test or the reference standard.	Not applicable
Discussion		
26. Limitations	Study limitations, including sources of potential bias, statistical uncertainty and generalizability.	Discussion; paragraph 4
27. Implications for Practice	Implications for practice, including the intended use and clinical role of the index test.	Discussion; paragraph 1-3 & Conclusion
Other information		
28. Registration	Registration number and name of registry.	Not applicable
29. Protocol	Where the full study protocol can be accessed.	Not applicable
30. Funding	Sources of funding and other support; role of funders.	Conflicts of Interest and Source of Funding & Acknowledgments

Table S1: ROC analysis of APTT (seconds and preoperative ratio) for the predefined target conditions of anti-Xa activity (<0.1, <0.2, and <0.3 IU/mL): AUCs, 95% CIs, and Youden cut-offs with 95% CIs

Comparators	Target anti-Xa activity (IU/mL)	AUC	AUC 95%CI	Cut-off	Cut-off 95%CI
APTT (seconds)	<0.1	0.493	0.432 – 0.750	45.05	33.75 – 62.80
	<0.2	0.618	0.464 – 0.988	46.25	33.75 – 80.70
	<0.3	0.649	0.400 – 0.948	33.75	33.75 – 46.75
APTT (ratio)	<0.1	0.520	0.444 – 0.770	1.61	0.798 – 1.995
	<0.2	0.759	0.416 – 1.000	2.00	1.143 – 2.428
	<0.3	0.675	0.381 – 1.000	2.00	1.161 – 1.995

APTT, activated partial thromboplastin time; AUC, area under the ROC curve; CI, confidence interval.

APTT (ratio) = APTT at sampling ÷ preoperative APTT. Cut-offs were determined by Youden's index, and 95% CIs for AUCs and cut-offs were obtained by bootstrap resampling (1,000 iterations).

Table S2: Pairwise comparisons of AUCs between ΔR and APTT (seconds and ratio) at predefined anti-Xa targets (<0.1, <0.2, and <0.3 IU/mL) using DeLong's test

Comparisons	Target anti-Xa activity (IU/mL)	ΔR AUC	Comparators AUC	p value	95%CI (diffence in AUCs)
ΔR vs APTT (seconds)	<0.1	0.934	0.493	< 0.001	0.225 – 0.658
	<0.2	0.959	0.618	0.069	-0.027 – 0.709
	<0.3	0.921	0.649	0.327	-0.272 – 0.816
ΔR vs APTT (ratio)	<0.1	0.934	0.520	< 0.001	0.209 – 0.620
	<0.2	0.959	0.759	0.159	-0.079 – 0.479
	<0.3	0.921	0.675	0.389	-0.313 – 0.804

APTT, activated partial thromboplastin time; AUC, area under the ROC curve; CI, confidence interval.

Two-sided p values. The difference was calculated as AUC(ΔR) − AUC(comparator), and its 95% CI was computed by DeLong's method. APTT ratio = APTT at sampling ÷ preoperative APTT. Primary target: <0.1 IU/mL; <0.2 and <0.3 IU/mL are secondary.

Figure S1. Flowchart of participant enrollment. Of the 65 consecutive patients aged <18 years who received UFH in the PCICU (July 5, 2024–May 31, 2025), 6 were excluded due to heparin discontinued before sampling; therefore, 59 were included in the analysis.

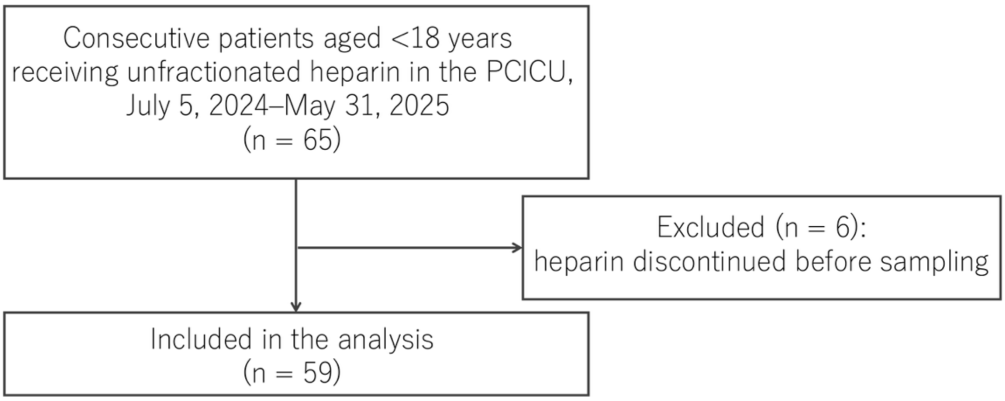

Figure S2. ROC curves for ΔR at different secondary targets.

(A) Target: anti-Xa < 0.2 IU/mL: AUC 0.959 (95% CI 0.893–1.000); cut-off 7.55 min (95% CI 6.90–12.95).
(B) Target: anti-Xa < 0.3 IU/mL: AUC 0.921 (95% CI 0.842–0.982); cut-off 7.55 min (95% CI 6.90–8.85).
ΔR = CK-R − CKH-R (min).

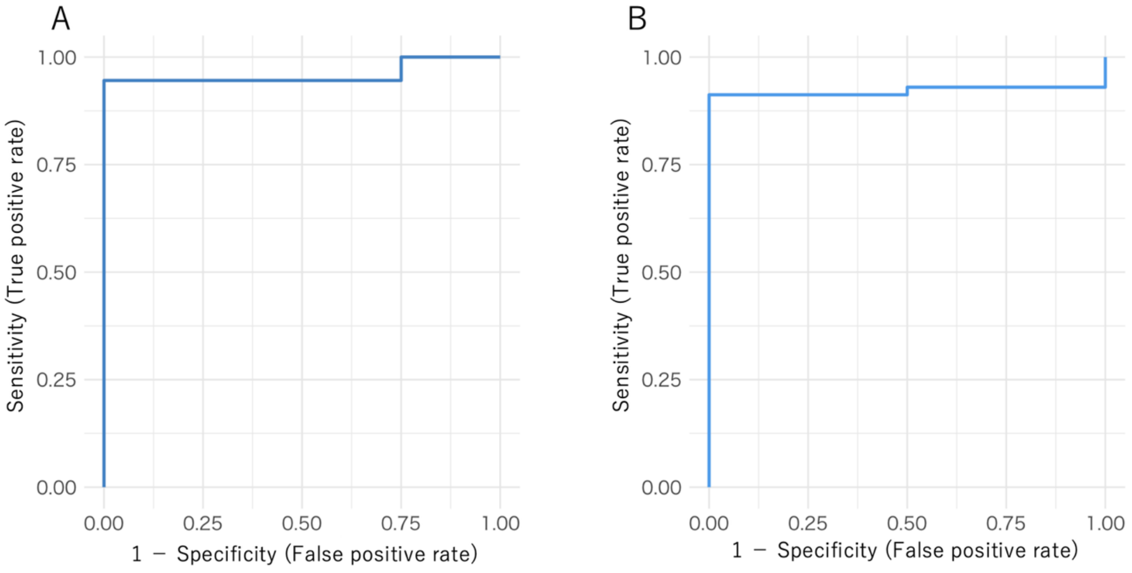

Figure S3. ROC curves for APTT measures at each target.

(A–C) Target APTT values (seconds): (A) anti-Xa < 0.1 IU/mL AUC 0.493 (95% CI 0.432–0.750), cut-off 45.05 s (95% CI 33.75–62.80); (B) anti-Xa <0.2 IU/mL AUC 0.618 (0.464–0.988), cut-off 46.25 s (33.75–80.70); (C) anti-Xa <0.3 IU/mL AUC 0.649 (0.400–0.948), cut-off 33.75 s (33.75–46.75).
(D–F) Target APTT ratios: (D) anti-Xa <0.1 IU/mL AUC 0.520 (0.444–0.770), cut-off 1.61 (0.798–1.995); (E) anti-Xa <0.2 IU/mL AUC 0.759 (0.416–1.000), cut-off 2.00 (1.143–2.428); (F) anti-Xa <0.3 IU/mL AUC 0.675 (0.381–1.000), cut-off 2.00 (1.161–1.995).
APTT ratio = APTT at sampling ÷ preoperative APTT.

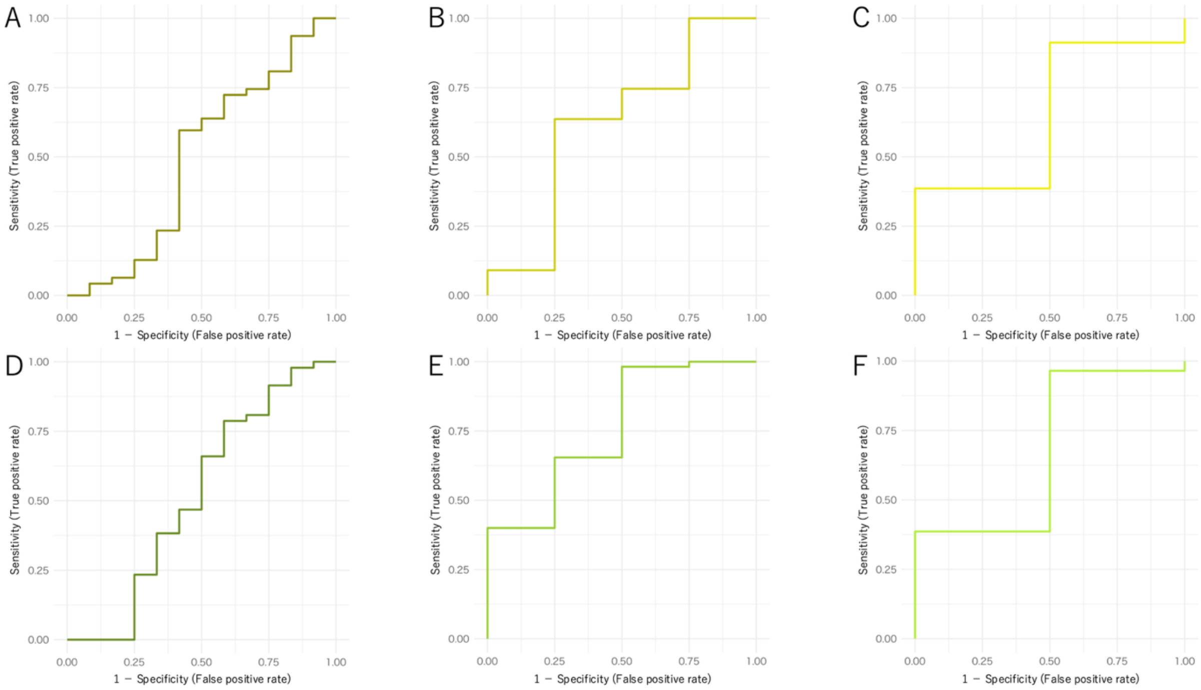

Figure S4. Scatter plot and ROC curve in the prespecified sensitivity analysis restricted to measurements with antithrombin activity ≥ 60% (n = 50).

(A) Scatter plot

The solid line represents the fitted simple linear regression (anti-Xa = 0.0126 + 0.0168 × ΔR; $R^2 = 0.4333$; residual SD = 0.0654), and the shaded band indicates the 95% confidence interval.

(B) ROC curve

For the primary target condition (anti–Xa <0.1 IU/mL), the AUC was 0.932 (95% CI, 0.827–1.000) with an optimal cut-off of 5.6 min (bootstrap 95% CI, 4.35–6.35).

ΔR = CK-R − CKH-R (min).

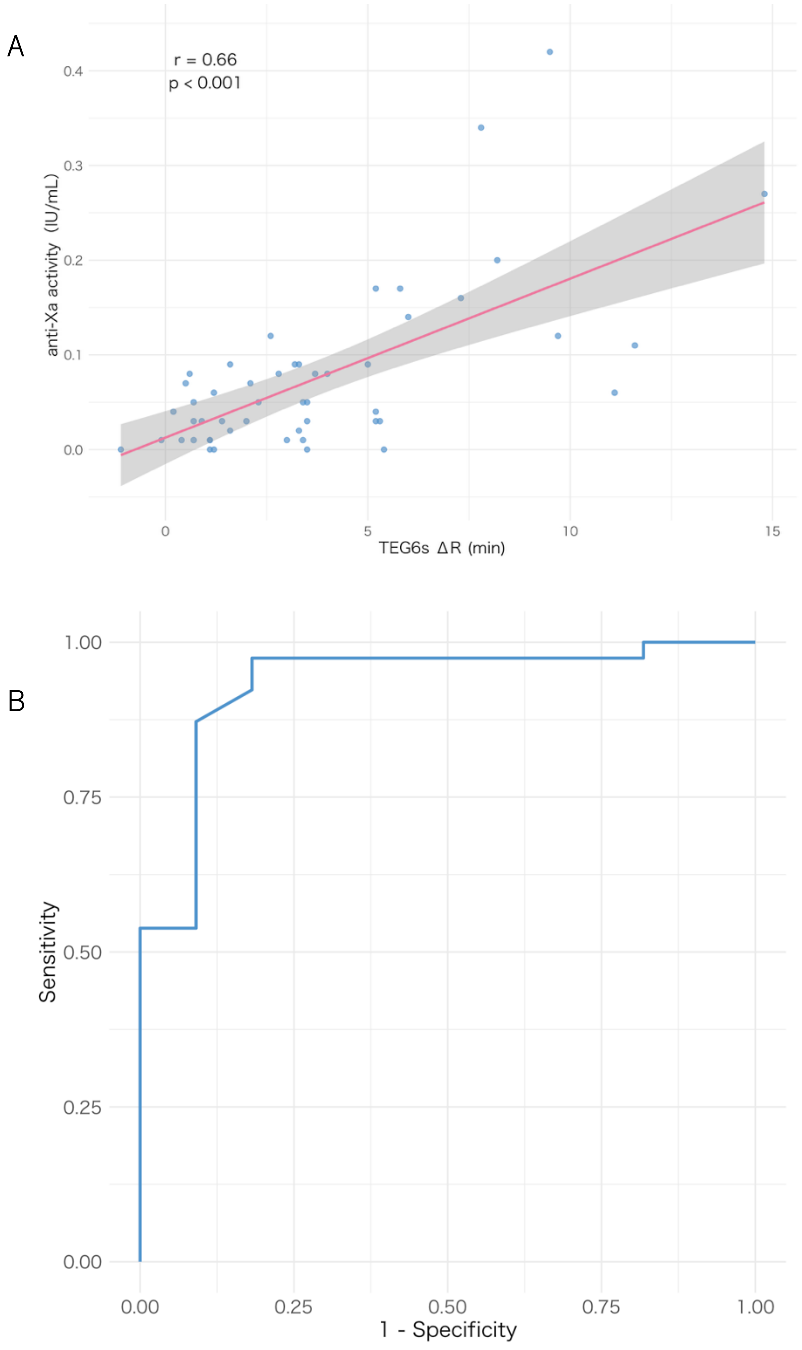

Supplement: Supplementary file 1 [file cc9-8-e1400-s001.pdf]
